# In Memoriam

**Published:** 1878-02

**Authors:** 


In Memoriam.—Dr. L. P. Yandell, Sr., of Louisville, Ky., is no more! This patriarch in medicine died at his home, in Louisville, on the 4th of February, 1878, full of years and full of honors.

Says the Louisville *Medical News:* "Dr. Yandell died on the 4th of February, after a short illness with pneumonia. He was born July 4, 1803, and was consequently in his seventy-fifth year when he died. He was a native of Tennessee. * * * Dr. Yandell attended his first course of lectures in the Transylvania University, at Lexington, his second course in the University of Maryland, at Baltimore, where he graduated in 1825. In 1831 he was called to the chair of Chemistry in the Transylvania College, which position he held for six years, when (1837) he came to Louisville and assisted in the organization of the Medical Institute, which subsequently became the medical department of the University of Louisville. He filled, at different times, in the institution, the chairs of Chemistry, Materia Medica and Physiology."

The following report of a committee appointed by the Atlanta Academy of Medicine, was read and unanimously adopted at a regular meeting of the Academy, on the 25th of February, 1878:

The undersigned, a committee of the Atlanta Academy of Medicine, appointed to prepare a suitable tribute to the memory of the late Prof. Lunsford P. Yandell, have had the same under consideration, and ask leave to report:

That it is with unaffected sorrow we are called upon to record the death of this eminent man. He died peacefully, in the city of Louisville, Ky., on the fourth day of February, instant, surrounded by his descendants to the fourth generation, in the full enjoyment of his just fame honorably worn to old age; and, in his varied character of teacher, practitioner of medicine, author, minister of the Gospel, and citizen, he has gone to his final account, without a stain upon his character, or an enemy to asperse his good name.

Dr. Yandell began his career as a medical teacher in the medical department of Transylvania University, at Lexington, Ky. About the year 1825 he was associated with the great Dudley, the father of surgery in the West and South, and McDowell, the first man who removed an ovarian tumor. Also, with Eberle, Caldwell, and others whose names are well known in medical annals. Under their championship the school at Lexington became as famous for medical teaching as those of Philadelphia, New York and Boston. The rapid growth of Louisville, Ky., developed a better field for medical teaching, and Dr. Yandell with most of his colleagues were elected to chairs in the University school, in 1837. He continued in this school until 1857, when the ill health of his family caused his removal to Memphis, where he had been called to take the chair of Theory and Practice of Medicine, in Memphis Medical College. This he resigned in 1862, and sought a higher plane of usefulness as minister of the Gospel. After the war he returned to Louisville, where necessity compelled him to resume his profession, except on Sundays, which he gave to the Church.

Dr. Yandell was an educated man, and loved books and literature. He was also a man of large capacity, and read books to purpose. He has written more voluminously than any of his cotemporaries. He was three times, we believe, the editor of different medical journals, and left his imprint broad and deep upon the medical literature of the country.

It was the good fortune of some of the committee to have known him long, and it is their opinion that a nobler type of our race is rarely if ever vouchsafed to humanity. The ideal of the poet and painter were blended in his symmetrical character and personelle. Never too early; never too late; never wishing to lead, yet always in the lead. Stern to stubbornness where duty demanded; yet gentle as the gentlest, where moral sense interposed. A personal integrity above temptation—a sacred regard for truth that nothing could swerve; a courage that nothing could daunt; a catholic embrace of all that contributes most to the wisdom, happiness and dignity of man.

The committee conclude with the following resolution:

*Resolved*, That the Secretary be requested to forward a copy of these proceedings to the family of Dr. Lunsford P. Yandell, as a token of the esteem in which he was held by the Atlanta Academy of Medicine.

L. G. Alexander, M.D.,
Jno. M. Johnson, M.D.,
W. F. Westmoreland, M.D.

On motion, unanimously adopted.

Jas. B. Baird, M.D., Sec'y.

